# Publication Rates and Inconsistencies of the Abstracts Presented at the National Anatomy Congresses in 2007 and 2008

**DOI:** 10.4274/balkanmedj.2016.0360

**Published:** 2017-01-05

**Authors:** İlke Ali Gürses, Özcan Gayretli, Başak Gürtekin, Adnan Öztürk

**Affiliations:** 1 Department of Anatomy, İstanbul University İstanbul Faculty of Medicine, İstanbul, Turkey; 2 Department of Biostatistics and Medical Informatics, İstanbul University İstanbul Faculty of Medicine, İstanbul, Turkey

**Keywords:** Congresses, meeting abstracts, publications, publication rate, inconsistencies

## Abstract

**Background:**

Despite significant efforts made for, most abstracts presented during a meeting do not proceed and publish as a manuscript in scientific journals.

**Aims:**

To investigate publication rates of national anatomy congresses.

**Study Design:**

Descriptive study.

**Methods:**

All abstracts presented at two annual meetings in 2007 and 2008 were extracted. PubMed and Google Scholar database search used for publication history. Presentation and study types, publication rates and mean publishing times were evaluated. Inconsistency rates between meeting abstract and final published article were also considered.

**Results:**

Among 342 abstracts, 195 (57%) were followed by a full-text article. Publication rates for oral and poster presentations were 75% and 52.2%, respectively. The mean publication time was 23.7±23 months. Overall, 89.2% of the articles were published within 5 years. There were no inconsistencies in 50 (25.6%) articles, while 145 (74.4%) had inconsistencies compared to the abstracts presented at the congress. Getting adequate information for 45 (23.1%) articles was not possible. There was no standard reporting format for the abstracts.

**Conclusion:**

Our study shows that, overall publication rates for abstracts presented at national anatomy meetings were higher than those presented at national meetings for clinical specialties.

Congresses and symposia are necessary media for sharing scientific experiences, collaborating with colleagues and finding new ideas for future scientific research (1,2). Despite these opportunities, many abstracts presented at scientific meetings are not published as full-text articles in peer-reviewed journals (3). Therefore, one could surmise that the publication rate may represent the scientific quality of a given meeting (1,2,3,4).

There are numerous studies that evaluate the publication rates of abstracts presented at scientific meetings and congresses in medical literature for various clinical specialties and subspecialties (1,2). The publication rates for these international meetings vary between 0% and 82% (2). As for national meetings, previous studies focused only on clinical medical sciences with a publication rate ranging between 9.4% and 29.5% (4,5,6,7,8,9,10,11,12). Additionally, we were unable to find studies that evaluated the publication rates of basic medical sciences. With this study, we aimed to investigate the publication rates of abstracts presented at National Anatomy congresses and evaluate any inconsistencies that were present between the congress abstract and the final published article.

## MATERIALS AND METHODS

Yalçınkaya and Bagatur (8) evaluated previous studies on national orthopaedic meetings and concluded that 5 years is sufficient for drawing a conclusion on publication rate. Therefore, we decided that January 2010 would be our cut-off date. We included the national congresses of 2007 and 2008 in our study, because in 2009 a national meeting was not organized.

We obtained the presented abstracts from both meetings via Anatomy - International Journal of Experimental and Clinical Anatomy (13,14). After obtaining the abstracts, first we categorized them according to presentation type (oral or poster) and study type (clinical anatomy, experimental studies, case reports, anatomy education, anthropometric studies, anatomy history, anatomical terminology and reviews). Clinical anatomic studies were performed on human materials including cadavers, dry bones, patients and radiologic images. Experimental studies included laboratory animal studies that required Animal Experimentations Ethics Board approval. Educational studies included graduate (medical, dentistry and allied health professions) and postgraduate education.

We used the electronic search engines PubMed (National Library of Medicine, Bethesda, Maryland, USA) (15) and Google Scholar (Google Inc., Mountain View, California, USA) (16) to determine whether an abstract was published as a full-text scientific article. We performed the initial search on Google Scholar to evaluate the abstracts, because it has a wider coverage for scientific articles (17). After finding the published abstract, we cross-checked the full title of the article on PubMed and confirmed the final publication on the MEDLINE® database. If we could not verify an article in PubMed, we evaluated whether the journal was indexed in the Master Journal List (Thomson Reuters, New York, USA) (18) or TUBİTAK ULAKBİM (Cahit Arf Bilgi Merkezi, Ankara, Turkey) (19) databases. For the search algorithm, first we used the full title of the abstract. If we could not find any results, we repeated the search with keywords from the abstract title and the surname of the first author with the Boolean operator AND. If no results were found, we used subsequent authors or combinations of author names. When we came across more than one article originating from one abstract, we accepted the earliest published article, in order to prevent iteration.

We identified the time of publication as the time lag between the congress and the final publication of articles in months. We included the studies that are published before the congresses and accepted their publication time as minus (-) months.

We recorded the name of the journals that the articles published. To evaluate the impact factor (IF) of the journals, we calculated the average IF of each journal between 2010 and 2014 via Journal Citation Reports^®^ (Thomson Reuters, New York, USA) (20) as of August 2015.

We compared the presented abstract and the final published article to evaluate any inconsistencies. We modified the methodology of Bhandari et al. (1) and Yalçınkaya and Bagatur (8) and categorized the inconsistencies as minor and major. We excluded parameters regarding patient treatment (e.g. primary and secondary outcome measures). Minor inconsistencies included changes to the title of the study, the number of authors, the first author name and names of other authors. Major inconsistencies included changes to the objective/hypothesis of the study, the sample size, the statistical methods used and the results. We considered the congress abstracts that did not provide clear data (e.g. objective, sample size or results) to be inconsistent. As for changes to the author list, we also evaluated whether any authors of the meeting abstract had been deleted.

We used statistical analysis software (SPSS v.21, IBM Corp., New York, USA, 2012) to evaluate our results. Comparison of publication rates per year, presentation types and study topics was evaluated with chi-square analysis. We used the Mann-Whitney U test to compare the mean publication time and average IF for presentation types. We used the Kruskal-Wallis test to compare the mean publication time of the articles with/without inconsistencies.

## RESULTS

A total of 342 abstracts were presented at the 2007 (189 abstracts) and 2008 (153 abstracts) National Anatomy congresses. The total number of oral and poster presentations was 72 (21.1%) and 270 (78.9%), respectively.

Three abstracts were followed by seven articles. The articles that were published earliest were accepted as final publications and the remaining four were excluded from the study to avoid iteration. As of August 2015, 195 abstracts were followed by a full-text article with a publication rate of 57%. Among these, 115 (33.6%) were published in MEDLINE-indexed journals. Twenty-one (6.1%) abstracts were published in journals indexed in Science Citation Index - Expanded but not in the MEDLINE database. Finally, 59 (17.3%) abstracts were published in national journals indexed in the TUBİTAK ULAKBİM database or international journals that are not indexed in Science Citation Index - Expanded. There was no statistically significant difference for publication rates between 2007 and 2008 meetings (p=0.342). The publication rates for oral and poster abstracts published in MEDLINE-indexed journals were 54.2% (39/72) and 28.1% (76/270), respectively. The publication rates for all oral and poster presentations were 75% (54/72) and 52.2% (141/270), respectively. Oral presentations had a statistically higher publication rate than poster presentations (p=0.001). Table 1 summarizes the publication rates per congress year.

The mean publication time for all abstracts was 23.7±23 months. Twenty (10.2%) abstracts were published prior to the meetings with a mean publication time of -9.1±-6.1 (min: -22, max: -1) months. The number of published abstracts after congress per year was 52 (26.7%) for the first year, 48 (24.6%) for the second year, 24 (12.3%) for the third year, 21 (10.8%) for the fourth year, 9 (4.6%) for the fifth year and 21 (10.8%) for more than 5 years. Most of the abstracts (89.2%) were published within the first 5 years. Table 2 outlines the number of abstracts published per year. The mean publication times for oral and poster presentations were 22.7±21.4 and 24±23.6 months, respectively. The difference in mean publication time for presentation types was not statistically significant (Z=0.261, p=0.794). Table 3 summarizes the mean publication times for abstracts in terms of presentation and study topics.

One hundred and sixty-six (48.5%) abstracts were related to clinical anatomy, 76 (22.2%) were experimental, 57 (16.7%) were case reports, 22 (6.4%) were anthropometric, 8 (2.3%) were related to anatomy education, 6 (1.8%) to history, 4 (1.2%) concerned anatomical terminology and 3 (0.9%) were review studies. Table 3 outlines the publication rates according to presentation type and study topic.

Final articles were published in 106 different journals. The six most widely preferred journals were: Surgical and Radiologic Anatomy (12-6.2%), Clinical Anatomy (9-4.6%), Journal of Craniofacial Surgery (9-4.6%), Turkish Journal of Medical Sciences (8-4.1%), Fırat Medical Journal (7-3.6%) and International Journal of Morphology (7-3.6%).

The average IF of the journals indexed in the MEDLINE database for the 2010 to 2014 period for total, oral and poster presentations was 1.312±0.752, 1.581±0.770 and 1.173±0.709, respectively. The difference between the mean IF of the journals that published oral and poster presentations was statistically significant (Z=3.034, p=0.002). Oral presentations tended to be published in journals with a higher mean IF.

We could not find any inconsistencies in 50 (25.6%) articles. One hundred and forty-five (74.4%) articles had inconsistencies compared to the congress abstract. Among these, 78 (40%) had only minor, 6 (3.1%) had only major and 61 (31.3%) had both minor and major inconsistencies. Minor inconsistencies included changes to the title in 119 (61%) articles, the number of authors in 74 37.9 (%), the first author’s name in 30 (15.4%) and the number of other authors in 84 (43.1%) articles. Major inconsistencies included changes to the study design/hypothesis in 21 (10.8%) articles, the sample size in 52 (26.7%), the statistical method in 58 (29.7%) and the results in 49 (25.1%) articles. Table 4 outlines all inconsistencies.

The mean publication times for articles with no, only minor, only major, and both minor and major inconsistencies were 16.9±24.3, 22.2±20.2, 30.8±27.1 and 31.3±23.5 months, respectively. Articles with no inconsistencies had a statistically significantly shorter mean publication time (χ2=17.120, p<0.001).

We found that 45 abstracts (23.1%) did not provide sufficient data on the study. In 10 (5.1%) abstracts, the sample size was not mentioned. Forty-two (21.5%) abstracts did not define the statistical method(s) used. Eleven (5.6%) abstracts did not report results.

Additionally, we detected author deletions between the meeting abstract and published article in 35 (18%) studies.

## DISCUSSION

Although there are numerous articles that evaluated the publication rates of abstracts presented at scientific meetings for different clinical specialties and subspecialties, we were unable to find a study that investigated this topic for basic medical sciences in international and national literature. Therefore, we compared our results with other national congresses.

National meetings for clinical sciences had a publication rate of 29.5% for orthaopedics (8), 28.6% for reproductive endocrinology and infertility (11), 21.7% for rheumatology (6), 13.2% for dermatology (7), 11.8% and 9.4% for radiology (4,10) and 5.7% for general surgery (5). For total (57%), oral (75%) and poster (52.2%) presentations, anatomy congresses had a higher publication rate than any other clinical sciences (Table 5). Nevertheless, we believe that the number of presented abstracts (161 to 4413) and the number of congresses evaluated (1 to 10) in these studies may have an effect on publication rates (4,5,6,7,8,9,10,11,12). There is another possible reason why the publication rates for some clinical specialties are dramatically lower. Case report presentations represent 45.2% to 68.6% of all abstracts presented in the fields of general surgery, radiology, orthopaedics and dermatology (4,5,7,8,10). Most of these studies report the lowest publication rates (between 5.7% and 13.2%) at Turkish national congresses. At anatomy congresses, the majority of the abstracts were experimental (272/342, 79.5%) and case reports represented only 16.6% of all presentations. A similar trend was present at reproductive endocrinology and rheumatology congresses where clinical and experimental studies represented the majority of presentations (6,11). Literature shows that basic research studies are more likely to be published as articles (2,3). The main reason for this discrepancy is that clinical studies, apart from randomized controlled trials, have different study designs (e.g. case reports) that are often not published (3).

The publication time for abstracts presented at national meetings varied from 14.9 months to 30.72 months. The mean publication time (23.7 months) for abstracts presented at anatomy congresses was concordant with those presented at clinical meetings. Most of the congress abstracts (89.2%) were published as an article within the first 5 years of the presentation. This finding supports the results of Yalçınkaya and Bagatur (8) indicating that a 5-year period is sufficient for drawing a conclusion from a given congress. We found that 20 abstracts were published before the meetings. There are only a few articles in the literature reporting publication prior to congresses (8,21,22). Additionally, previous reports did not provide an explanation for this. We did not retrospectively evaluate or ask the corresponding authors about the reasons either. One might consider this acceptable if the study had been concluded after the previous meeting and subsequently published before the next congress. Despite this, we found that some studies had been published as a full-text article nearly 2 years (-22 months) prior to their presentation at a congress. Therefore, it remains unclear why these authors chose to present a study after its publication as an article.

We found that the most widely preferred journals were Surgical and Radiologic Anatomy, Clinical Anatomy, Journal of Craniofacial Surgery, Fırat Medical Journal and Turkish Journal of Medical Sciences. After evaluating the published articles on human anatomy between 2000 and 2014, Tellioğlu et al. (23) reported that Surgical and Radiologic Anatomy, Clinical Anatomy and Journal of Craniofacial Surgery were the journals most frequently preferred by Turkish anatomists. Although our results are similar, we believe that the study of Tellioğlu et al. (23) is more reliable in terms of including published articles within a wider time interval.

We observed that the majority of the articles (74.4%) had changes from the congress abstract. This finding is similar to that from other national congresses (8). In 61% of published abstracts, the title was changed. This rate is higher than in previously reported studies (1,8). Although any changes to a scientific study seem concerning, authors do make acceptable changes (e.g. changing the title, reorganizing the abstract) to their articles prior to submission in order to increase the impact of their research or because changes are suggested during the peer-review process by referees mostly regarding the title of the paper (8,24). As for author names, our results are in concordance with the study of Yalçınkaya and Bagatur (8). They suggested that adding new authors to a study is controversial, especially without changing the study itself. This is called “Ghost and Honorary Authorship” (25,26). We partly disagree with this point of view. According to the International Committee of Medical Journal Editors (ICMJE) (27), performing the study (design, data acquisition, analysis and interpretation) is one criterion for authorship. This means that any individual who meets the second criterion (drafting or revising the intellectual content of the work), even after the presentation of the work at a meeting, should be considered an author. Nevertheless, it is nearly impossible for an outsider to identify a researcher as a real or ghost author. Additionally, author deletions from congress abstracts could be considered a drawback as well. Ersoy et al. (11) reported that there were author deletions from the published articles in 39.2% of the meeting abstracts in the field of reproductive endocrinology and infertility. Similarly, we found author deletions in 20% of the published articles. Although we believe author deletions are controversial, there may be some exceptions. First, many journals limit the number of authors in original articles and case reports. In these cases, authors could be deleted after providing consent. Nevertheless, choosing another journal without author limits remains an alternative. Secondly, the presented abstract may be a preliminary study. After obtaining constructive criticism during the congress, the researchers may have conducted a new study by expanding or changing their samples. Therefore, presented and published studies become two different studies. This may be the case for younger researchers who attend congresses for scientific mentoring from senior researchers.

Frequent major inconsistencies included changes to the sample size (26.7%), statistical methods (29.7%) and results (25.1%). Our results are similar to those of previous studies (8). It should be kept in mind that these three topics are closely associated with the sample size at the centre. A change in the sample size might change the statistical method used, and this affects the results. Another reason may be methodological flaws that were corrected during the peer-review process. Lastly, as discussed above, these studies could be preliminary studies. National meetings in particular are excellent opportunities for young researchers to present their work and obtain scientific feedback from senior researchers. We think that inconsistencies between congress abstracts and published articles from preliminary studies should be expected.

We found that 23.1% of presented abstracts did not report data including sample size, statistical methods and results. We accepted all abstracts with uncertain information as inconsistent. Providing inadequate data seems to be a common problem for meeting abstracts (8) and full-text articles (28). Although inadequate reporting is unacceptable for published articles, congress abstracts are contentious. Nevertheless, we suggest important information should be reported in meeting abstracts in order to set an example of good scientific practice for younger researchers. We suggest the congress abstracts should be structured and clear, and provide basic data. These data should include hypothesis, the sample size, the statistical methods used and the results supported with statistical significance. For preliminary studies that could not provide these data, we suggest including the term “preliminary study” within the title.

Our study has several limitations. We were unable to find any studies in international and national literature that provide publication rates for a basic medical science. Therefore, we compared our results with national congresses for different clinical specialties and subspecialties. Another limitation was that our cross-sectional study evaluated only two consecutive congresses. A longer time interval may have a more precise result for evaluating the publication rates of National Anatomy meetings. Finally, we were unable to explain why some of the studies were presented after their publication.

In conclusion, we think that further sectional studies on basic medical sciences should be performed regarding national and international congresses for comparing results. Repeating these studies for selected time periods may be helpful in improving the scientific content of a given specialty. Finally, we observed that abstracts presented at anatomy congresses did not have a standard reporting format. In order to improve this inadequacy, we suggest implementing the STROBE (29) checklist for reporting congress abstracts.

## Figures and Tables

**Table 1:**
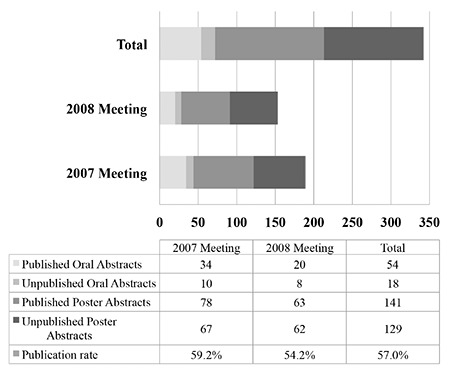
Publication rate per meeting

**Table 2:**
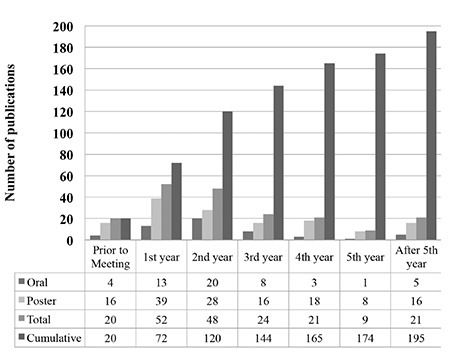
Number of published abstract per year

**Table 3:**
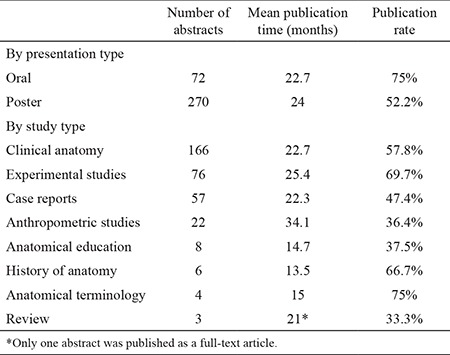
Mean publication times and publication rates for presentation and study types

**Table 4:**
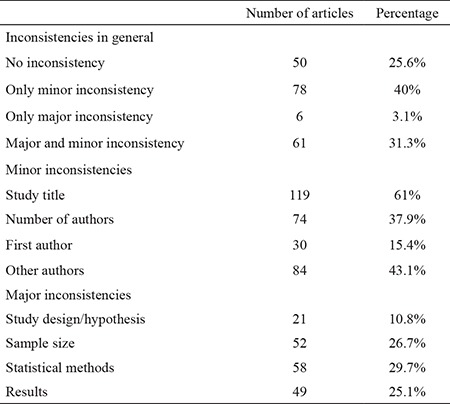
Inconsistencies between congress abstracts and full-text articles

**Table 5:**
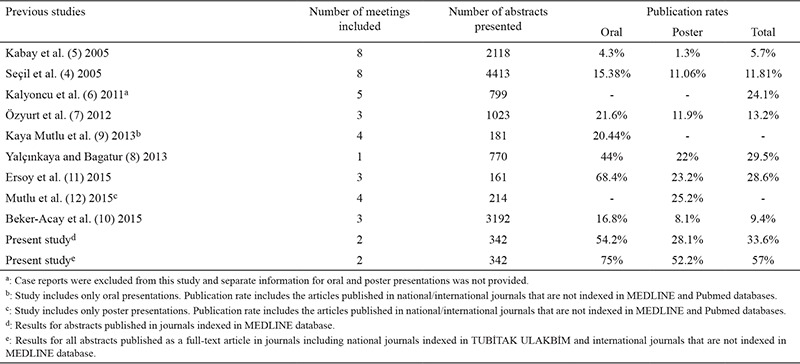
Studies on publication rates of different national congresses
